# Upregulation of TrkB Promotes Epithelial-Mesenchymal Transition and Anoikis Resistance in Endometrial Carcinoma

**DOI:** 10.1371/journal.pone.0070616

**Published:** 2013-07-30

**Authors:** Wei Bao, Haifeng Qiu, Tingting Yang, Xin Luo, Huijuan Zhang, Xiaoping Wan

**Affiliations:** 1 Department of Obstetrics and Gynecology, International Peace Maternity and Child Health Hospital Affiliated to Shanghai Jiao Tong University School of Medicine, Shanghai, China; 2 Department of Obstetrics and Gynecology, Shanghai First People’s Hospital Affiliated to Shanghai Jiao Tong University School of Medicine, Shanghai, China; 3 Department of the Centre of Research Laboratory, International Peace Maternity and Child Health Hospital Affiliated to Shanghai Jiao Tong University School of Medicine, Shanghai, China; 4 Department of Pathology, International Peace Maternity and Child Health Hospital Affiliated to Shanghai Jiao Tong University School of Medicine, Shanghai, China; Florida International University, United States of America

## Abstract

Mechanisms governing the metastasis of endometrial carcinoma (EC) are poorly defined. Recent data support a role for the cell surface receptor tyrosine kinase TrkB in the progression of several human tumors. Here we present evidence for a direct role of TrkB in human EC. Immunohistochemical analysis revealed that TrkB and its secreted ligand, brain-derived neurotrophic factor (BDNF), are more highly expressed in EC than in normal endometrium. High TrkB levels correlated with lymph node metastasis (*p*<0.05) and lymphovascular space involvement (*p*<0.05) in EC. Depletion of TrkB by stable shRNA-mediated knockdown decreased the migratory and invasive capacity of cancer cell lines in vitro and resulted in anoikis in suspended cells. Conversely, exogenous expression of TrkB increased cell migration and invasion and promoted anoikis resistance in suspension culture. Furthermore, over-expression of TrkB or stimulation by BDNF resulted in altered the expression of molecular mediators of the epithelial-to-mesenchymal transition (EMT). RNA interference (RNAi)-mediated depletion of the downstream regulator, Twist, blocked TrkB-induced EMT-like transformation. The use of in vivo models revealed decreased peritoneal dissemination in TrkB-depleted EC cells. Additionally, TrkB-depleted EC cells underwent mesenchymal-to-epithelial transition and anoikis in vivo. Our data support a novel function for TrkB in promoting EMT and resistance to anoikis. Thus, TrkB may constitute a potential therapeutic target in human EC.

## Introduction

Endometrial carcinoma (EC) is the most common cancer of the female genital tract. In the United States, approximately 49,500 new cases of EC will be diagnosed in 2013, and 8,200 deaths are expected [1]. Although most EC is diagnosed at an early stage, 30% of all cases are still diagnosed at later stages, which correlates with decreased survival rates [2]. Women with certain histological subtypes, high-grade lesions, deep invasion into the uterus, a tumor that extends into the cervix, or a tumor that has spread to the lymphatic system, the blood vessels or outside the uterus are at highest risk of recurrence [3]. The current treatments for EC include surgery and adjuvant radiotherapy or chemotherapy. However, only a select number of patients respond to these adjuvant therapies [4]. Therefore, it is critical to better understand the molecular mechanisms that drive EC.

Neurotrophic receptor tyrosine kinase B (TrkB), when bound to its high-affinity ligand, brain-derived neurotrophic factor (BDNF), plays an essential role in nervous system development [5]. Extensive evidence suggests that it is a key regulator of oncogenesis and tumor progression in various human cancers, including lung [6], breast [7], pancreatic [8], stomach [9], colon [9], [10], prostate [11], and ovarian [12]. Activation of the BDNF/TrkB pathway stimulates tumor cell proliferation [13], angiogenesis [14], and invasion and metastasis [15] and causes chemotherapy resistance [16]. TrkB signaling is also related to anoikis resistance, which inhibits cell death and provides protection against the metastatic spread of tumor cells [17]. Further research is needed to better understand how this cancer disseminates. Currently, the specific role of TrkB in human EC is unclear.

Epithelial-to-mesenchymal transition (EMT) is a well-described process whereby epithelial cells lose their polarity and cell-cell contacts, undergo a dramatic remodeling of the cytoskeleton, and acquire a migratory phenotype, subsequently activating a mesenchymal-like gene expression program [18]. In EC, myometrial invasion is considered one of the most important prognostic factors [19]. EMT has been extensively described in other types of cancer but has been poorly studied in EC [20].

Here, we describe, for the first time, the association between TrkB expression and EC clinical outcome. We identify a critical role for TrkB in promoting EMT and anoikis resistance in EC.

## Materials and Methods

### Ethics Statement

This study was approved by the Human Investigation Ethical Committee of International Peace Maternity & Child Hospital Affiliated Shanghai Jiao Tong University. The samples of endometrial carcinoma and normal endometrial tissues were collected after written informed consent from the patients. The animal research was carried out in strict accordance with the recommendations in the Guideline for the Care and Use of Laboratory Animals of China. The protocol was approved by the Committee on the Ethics of Animal Experiments of the Obstetrical and Gynecological Hospital affiliated Fu Dan University (Permit Number: SYXK (hu) 2008–0064). All efforts were made to minimize animal suffering.

### Tissue Collection

From August 2009 to April 2011, 110 cases of uterine endometrial cancer were obtained from patients who underwent surgery at the International Peace Maternity & Child Health Hospital affiliated with Shanghai Jiao Tong University School of Medicine. The stages and histological grades of these tumors were established according to the criteria of the Federation International of Gynecology and Obstetrics (FIGO) surgical staging system (2009) [21].The features of all EC tissue samples are provided in Table 1. Twenty-five normal endometrium samples were obtained from patients who underwent hysterectomies to treat other diseases, such as myoma or adenomyosis. Twenty endometrial atypical hyperplasia (EAH) tissues were also collected from patients who underwent hysteroscope examination because of irregular bleeding. None of the patients underwent hormone therapy, radiotherapy, or chemotherapy before surgery.

**Table 1:** The relationship between TrkB expression and clinicopathological features in EC.

Variable	No. patients (%)		TrkB expression		
	n	%	Negative	Positive	*p*
Total	110	100	28	82	
Age (years)					
≤50	15	13.6	3	12	0.756
>50	95	86.4	25	70	
FIGO stage					
Stage I	84	76.3	20	64	0.890
Stage II	11	10	3	8	
Stage III	12	11	4	8	
Stage IV	3	2.7	1	2	
Grade (Endometrioid, n = 94)					
G1	45	47.8	12	33	0.893
G2	40	42.5	9	31	
G3	9	9.7	2	7	
Histological type					
Endometrioid	94	85.5	23	71	0.548
Nonendometrioid	16	14.5	5	11	
Myometrial invasion					
<1/2	93	84.5	23	70	0.763
≥1/2	17	15.5	5	12	
Lymph node metastasis					
No	98	89.1	28	70	0.034*
Yes	12	10.9	0	12	
Lymphovascular space involvement					
No	82	74.5	25	57	0.045*
Yes	28	25.5	3	25	
ER expression					
Negative	27	24.5	8	19	0.614
Positive	83	75.5	20	63	
PR expression					
Negative	21	19.1	7	14	0.407
Positive	89	80.9	21	68	

* *p*<0.05 for the difference between TrkB expression in patients with and without lymph node metastasis or lymphovascular space involvement. An IHC staining score of ≥4 was considered positive for TrkB expression as defined in the Methods. No statistical differences were found regarding gender, stage, grading, histological type, myometrial invasion, and ER or PR status.

### Immunohistochemistry

All tissue sections (4 µm thick) were processed for hematoxylin and eosin (H&E) staining or immunohistochemistry (IHC) as previously described [12]. Briefly, after deparaffinization and dehydration, specimens were boiled in 10 mM sodium citrate buffer to unmask antigens. Specimens were then blocked and incubated with primary antibody overnight at 4°C. Antibody binding was detected using Envision reagents (Boster bio-engineering, Wuhan, PR China) according to the manufacturer’s instructions. Primary rabbit polyclonal antibodies against TrkB or phospho-TrkB (p-TrkB), and E-cadherin or N-cadherin were diluted 1∶100 and 1∶200, respectively. Primary rabbit monoclonal antibody against BDNF was diluted 1∶100. The specificity of the TrkB and p-TrkB antibodies was verified using paraffin embedded HEC-1B^NT^ and HEC-1B^sh−TrkB^ cell pellets as controls (Fig. S1).

For evaluation of BDNF, TrkB, and p-TrkB expression, staining intensity was scored as 0 (negative), 1 (weak), 2 (medium), or 3 (strong). The extent of staining was scored as 0 (0%), 1 (1%–25%), 2 (26%–50%), 3 (51%–75%), or 4 (76%–100%), according to the percentage of the positively stained areas in relation to the whole tumor area. The sum of the intensity score and the extent score was used as the final staining score (0–7) [22]. Tumors having a final staining score of 4 or higher were considered to be positive for expression. The results were assessed by two pathologists who were blinded to details regarding patient background.

### Reagents and Antibodies

Poly (2-hydroxyethyl methacrylate) (Poly-HEMA, P3932) was obtained from Sigma (Sigma-Aldrich; St. Louis, MO, USA). Recombinant human BDNF was purchased from Peprotech (Rocky Hill, NJ, USA). Rabbit polyclonal antibodies included: TrkB (ab51190), p-TrkB (ab51187), Twist (ab49254), E-cadherin (ab15148), N-cadherin (ab12221), and Vimentin (ab45939), all from Abcam Ltd. (Hong Kong, PR China). Rabbit monoclonal antibody BDNF (1087-1) was purchased from Epitomics (Burlingame, CA, USA). Rabbit monoclonal antibodies β-actin (4967S), and anti-rabbit IgG, HRP-linked antibody (7074P2) were obtained from Cell Signaling Technology (Shanghai, PR China). Mouse monoclonal antibody TrkB (sc-136990) was purchased from Santa Cruz Biotechnology. FITC-goat anti-mouse IgG secondary antibody (BA1101) and TRITC-goat anti-rabbit IgG secondary antibody (BA1090) was purchased from Boster bio-engineering (Wuhan, PR China).

### Cell Culture

Human endometrial cancer cell lines (Ishikawa, RL95-2, HEC-1B, KLE, AN3CA, and SPEC-2) were obtained from the Chinese Academy of Sciences Committee Type Culture Collection (Shanghai, China). Cells were maintained at 37°C in a humidified atmosphere containing 5% CO_2_ in Dulbecco’s modified Eagle’s medium (DMEM)/F12 (11030; Gibco, Auckland, NZ) supplemented with 10% fetal bovine serum (FBS) (16000-44; Gibco, Carlsbad, CA). The human epithelial ovarian cancer cell line OVCAR-3 was kindly provided by Professor Xiaohui Yu (Department of Obstetrics and Gynecology, Shanghai First People’s Hospital Affiliated to Shanghai Jiao Tong University School of Medicine, Shanghai, China). The cells were cultured in RPMI-1640 medium supplemented with 15% FBS as previously described [12].

### Vector Construction and Lentiviral Transduction

The human TrkB gene (GC-Z0711, GeneCopoeia; Guangzhou, PR China) was cloned into pLV.EX3d.P/puro-EF1A> IRES/eGFP using Gateway technology, according to the protocol (http://products.invitrogen.com/ivgn/product/12538120). Short hairpin RNAs (shRNAs) were inserted into the XhoI (D1094A, Takara; Dalian, PR China) and HpaI (D1064A, Takara; Dalian, PR China) sites of pLenti X1/puro. The shRNA oligo sequences are provided in Table 2.

**Table 2:** shRNA oligo sequences.

Oligo name	Oligo sequences
shnon target-F	TGCGCGCTTTGTAGGATTCGCTCGAGCGAATCCTACAAAGCGCGCTTTTTC
shnon target-R	TCGAGAAAAAGCGCGCTTTGTAGGATTCGCTCGAGCGAATCCTACAAAGCGCGCA
shNTRK2#1-F	TCCTAATATGTATTGGGATGTTCTCGAGAACATCCCAATACATATTAGGTTTTTC
shNTRK2#1-R	TCGAGAAAAACCTAATATGTATTGGGATGTTCTCGAGAACATCCCAATACATATTAGGA
shNTRK2#2-F	TGCGCTTCAGTGGTTCTATAACCTCGAGGTTATAGAACCACTGAAGCGCTTTTTC
shNTRK2#2-R	TCGAGAAAAAGCGCTTCAGTGGTTCTATAACCTCGAGGTTATAGAACCACTGAAGCGCA
shNTRK2#3-F	TATCGTGGCATTTCCGAGATTGCTCGAGCAATCTCGGAAATGCCACGATTTTTTC
shNTRK2#3-R	TCGAGAAAAAATCGTGGCATTTCCGAGATTGCTCGAGCAATCTCGGAAATGCCACGATA

Ishikawa cells were transduced with pLV.EX3d.P/puro-EF1A> IRES/eGFP (empty vector, EV) or pLV.EX3d.P/puro-EF1A> NTRK2>IRES/eGFP (TrkB) viral supernatant in the presence of 6 µg/ml polybrene (H9268, Sigma; St. Louis, MO, USA). Stable cell lines were established using puromycin (2 µg/ml). To generate cell lines expressing shRNAs, HEC-1B cells were infected with non-target (NT) or TrkB-specific shRNA lentiviral particles in six-well plates in the presence of polybrene (6 µg/ml). Cells were treated with puromycin (2 µg/ml) to generate stable TrkB knockdown clones, and several puromycin-resistant TrkB knockdown clones were harvested by ring selection. The levels of TrkB over-expression and knockdown were confirmed by RT-PCR and immunoblotting (Figs. S2 and S3).

### Preparation and Transfection of siRNA Targeting Twist

To inhibit the expression of endogenous Twist, we designed and prepared three HPLC-purified Twist siRNAs (#1, #2 and #3) according to the sequence of the Twist gene. A scrambled siRNA with no homology to any known human mRNA was used as negative control. siRNA oligonucleotide duplexes were synthesized by Genephama Biotech (Shanghai, China). The sequences of siRNA oligos are provided in Table 3.

**Table 3:** siRNA oligos.

Oligo name	Oligo sequences
sinon target-F	UUCUCCGAACGUGUCACGUTT
sinon target-R	ACGUGACACGUUCGGAGAATT
siTwist#1-F	GGUGUCUAAAUGCAUUCAUTT
siTwist#1-R	AUGAAUGCAUUUAGACACCTT
siTwist#2-F	GAGACCUAGAUGUCAUUGUTT
siTwist#2-R	ACAAUGACAUCUAGGUCUCTT
siTwist#3-F	GGUACAUCGACUUCCUCUATT
siTwist#3-R	UAGAGGAAGUCGAUGUACCTT

Cells were seeded in 6-well plates at 70–80% confluence and grown overnight before transfection. Transfection of Ishikawa^TrkB^ cells with the siTwist or non-target control (siRNA-NT) was accomplished using the lipfectamine2000 transfection reagent (Invitrogen, Carlsbad, CA, USA) according to the manufacturer’s instructions. The experiments were classified into three groups: the siTwist group, the siRNA-NT group, and the untreated group.

### Recombinant Human BDNF Treatment

For BDNF treatment, 1×10^6^ Ishikawa and 2×10^6^ RL95-2 cells were seeded onto each well of a 6-well plate and cultured in DMEM/F12 medium supplemented with 10% FBS overnight. The cells were treated with recombinant human BDNF (Peprotech, Rocky Hill, NJ, USA) in FBS free medium at a concentration of 100 ng/ml. For the control, an equal volume of double distilled water was added. Cells were harvested after 24 h and 48 h of treatment for extraction of RNA and protein, respectively. Cell proliferation and invasion assays were performed after 24 h treatment [23].

### Cell Proliferation and Soft Agar Colony Formation Assays

To assess cell proliferation, cells were plated into 96-well plates at 2,000 cells per well. At each indicated time, the number of metabolically active cells was measured by 3-(4,5-dimethylthiazol-2-yl)-2,5-diphenyltetrazolium (MTT) (Sigma; St. Louis, MO, USA) assay. Absorbance values were measured at 490 nm with a Spectra Max 190 microplate reader (BIO-RAD model 680; USA).

To assess colony formation in soft agar, DMEM (2 ml) containing 0.7% agarose (Ameresco; Solon, Ohio, USA) and 10% FBS was poured into six-well dishes. The layer was covered with a mixture of medium, and 100 trypsinized HEC-1B cells or 500 trypsinized Ishikawa cells were seeded in 0.42% low-melting point agarose. Colonies were visualized and counted under a microscope after 2 weeks. The experiments were repeated at least three times.

### Cell Migration and Invasion Assays

For wound healing experiments, cells (6×10^6^ per well) were seeded in six-well plates and allowed to adhere for 24 h. Cell monolayers were scarred with a sterile micropipette tip and incubated in serum-free medium for 24 h. For each sample, three defined areas were monitored, and photographs were taken at 0 h, 6 h, 12 h, and 24 h to measure the migration index–a value derived by calculating the distance traveled by the cell monolayer relative to the gap made using the pipette tip at 0 h [24] – for control, shRNA targeted or over expression cells.

For invasion assays, experiment were performed using 6.5 mm transwell chambers equipped with 8.0 um pore-size polycarbonate membranes (Corning; Glendale, Arizona, USA). In these assays, the upper chambers were first coated with 40 µl of Matrigel at a 1∶3 dilution (BD Biosciences) and incubated at 37°C for 2 h. Then 2×10^5^ cells were added to the top section of a Boyden chamber apparatus in 150 µl serum-free DMEM medium. 600 µl complete medium was added to the lower chamber. After 48 h of incubation, cells attached to the underside of the membrane were fixed with 4% paraformaldehyde. Cells were stained with 5% crystal violet and counted at 200-fold magnification. The migration and invasion assay experiments were repeated at least three times.

### Anchorage-independent Cell Growth/Anoikis Assay

For anchorage-independent culture, 1×10^6^ adherent cells were trypsinized and reseeded in flat-bottomed, six-well plates coated with Poly-HEMA in DMEM/F12 supplemented with 10% FBS. These suspension cells gradually formed increased numbers of cell aggregates. We hypothesized that these cell aggregates (cell spheroids with anchorage-independent culture) could maintain partial anti-anoikis capacity and enable the EC cells with metastatic potentials.

For anoikis assay, 1×10^6^ cells were plated into Poly-HEMA coated 6 well plates in complete medium. After anoikis induction for 24 h and 48 h, cells were washed and resuspended in 0.5 ml binding buffer, and annexin V-PE/7-AAD labeling performed according to the manufacturer’s protocol (BD Bioscience; Franklin Lakes, NJ, USA). Analysis was performed with a Beckman Coulter FC 500 counter (Beckman Coulter, Inc.). The percentage of apoptotic cells was calculated by adding the fraction of cells with early apoptotic cells (LR%) and late apoptotic cells (UR%). All data were repeated at least three times and were analyzed statistically for determination of significance with appropriate standard deviation.

### Immunofluorescence

Cell spheroids from suspension cells were adhered to glass coverslips for 4 h in a 5% CO_2_ humidified incubator at 37°C. Cells were then fixed in 4% paraformaldehyde and permeabilized with 0.2% Triton X-100. Next, cells were blocked in 5% normal goat serum in PBS with 0.2% Tween for 1 h. Cells were incubated with the primary antibody (mouse anti-TrkB 1∶100 and rabbit anti-Twist 1∶100) diluted in blocking buffer overnight at 4°C, which was followed by incubation with secondary antibodies coupled to FITC (1∶100) and TRITC (1∶100) for 1 h at room temperature. Nuclei were visualized by counterstaining with 496-diamidino-2-phenylindole (DAPI). Samples were then viewed using a laser scanning confocal microscope (Leica TCS-SP5 II).

### RNA Extraction and qRT-PCR

Total RNA was isolated using Trizol reagent (Invitrogen**,** Life Technologies; Shanghai, PR China) and reverse transcribed using a reverse transcriptase kit (Takara, Dalian, PR China). Gene expression detection was performed with SYBR green master mix (Takara, Dalian, PR China) on an ABI Prism 700 thermal cycler (Applied Biosystems, Foster City, CA, USA) according to the manufacturer’s instructions. Gene expression was calculated using the 2?(−ΔΔCt) formula. Primers were designed using Primer Express software, and are shown in Table 4. The experiments were repeated at least three times.

**Table 4:** Primer used for real-time PCR analysis.

mRNA	Size(bp)	Primer sequence
BDNF	165	Forward 5′-GACATCATTGGCTGACACTTTC-3′
		Reverse 5′-TCCAGCAGAAAGAGAAGAGGAG-3′
NTRK2(TrkB)	88	Forward 5′-GGGACACCACGAACAGAAGTA-3′
		Reverse 5′-ACCACAGCATAGACCGAGAGA-3′
Twist 1	148	Forward 5′-GTCCGCAGTCTTACGAGGAG-3′
		Reverse 5′-GTCTGAATCTTGCTCAGCTTGTC-3′
Snail	218	Forward 5′-TCCAGAGTTTACCTTCCAGCA-3′
		Reverse 5′-CTTTCCCACTGTCCTCATCTG-3′
CDH1(E-cadherin)	179	Forward 5′-TTGCTACTGGAACAGGGACAC-3′
		Reverse 5′-CCCGTGTGTTAGTTCTGCTGT-3′
CDH2(N-cadherin)	161	Forward 5′-GGAGACATTGGGGACTTCATT-3′
		Reverse 5′-TCCTGCTCACCACCACTACTT-3′
CTNNB1(β-catenin)	169	Forward 5′-TGCTGAAGGTGCTATCTGTCTG-3′
		Reverse 5′-TCCATCCCTTCCTGTTTAGTTG-3′
Vimentin	240	Forward 5′-TGCGTGAAATGGAAGAGAACT-3′
		Reverse 5′-TCAGGTTCAGGGAGGAAAAGT-3′
β-actin	151	Forward 5′-CAGCCATGTACGTTGCTATCCAGG-3′
		Reverse 5′-AGGTCCAGACGCAGGATGGCATG-3′

### Western-blotting Assays

Cells were harvested, and protein was extracted using Mem-PER Eukaryotic Membrane Protein Extraction Reagent (Pierce; Rockford, IL, USA) containing complete mini cocktail, NE-PER Nuclear and Cytoplasmic Extraction Reagents (Pierce; Rockford, IL, USA) with protease inhibitor cocktail. All extracts were lysed according to the protocol. Total protein content was determined by BCA protein assay kit (Pierce; Rockford, IL, USA). Membranes were blocked and then probed with antibodies against TrkB (1∶500), p-TrkB (1∶500), BDNF (1∶1000), Twist (1∶500), E-cadherin (1∶500), N-cadherin (1∶500) and Vimentin (1∶500). The data have been normalized to β-actin expression (1∶2000) by densitometry and statistical data from at least 3 experiments is graphed to provide additional validation of results.

### In vivo Fluorescence Imaging

Twenty 5–6 week-old female athymic nude mice (BALB/c; five mice per group) were obtained from Shanghai life science institute (Slac Laboratory Animal co., LTD, China). TrkB stable knockdown and over-expression cells (HEC-1B^sh−TrkB^ and Ishikawa^TrkB^) and their respective controls (HEC-1B^NT^ and Ishikawa^EV^) were confirmed to express eGFP. Intraperitoneal injection in nude mice was performed using 5×10^6^ HEC-1B cells or 1×10^7^ Ishikawa cells in 500 ml PBS. eGFP reporter imaging was performed to monitor the cell seeding on the days 3, 14, 21, and 28 after injection by using the NightOWL LB981 NC100 system (Berthold Technologies; Bad Wildbad, Germany). By the 28th day after inoculation, mice were humanely euthanized, and laparotomy was performed to resect and count any disseminated tumors of >1 mm in diameter. The volume of the tumor dissemination was measured by the sum of all disseminated tumors, and the tumor volume was calculated as (Rmax)×(R^2 ^min)/2.

### Terminal Deoxynucleotidyl Transferase Mediated dUTP Nick End Labeling (TUNEL) Assay

Formalin-fixed, paraffin-embedded sections of disseminated tumors from nude mice were analyzed using a TUNEL Apoptosis Assay Kit (Roche; Indianapolis, IN, USA). The assay was performed according to the instructions in the user’s manual. Total DNA was stained with DAPI, and apoptotic cells appeared green under a fluorescent microscope (Leica DMI 3000B).

### Statistical Analysis

All statistical analyses were done using Statistical Package for the Social Sciences (SPSS) software version 17.0 (Chicago, IL, USA). Data is represented as means with standard deviations (SD). Measurement data were assessed using an unpaired Student’s t test, or one-way ANOVA analysis for multiple comparisons. The χ^2^ test for 2×2 tables was used to compare categorical data. *P values <0.05 or **P values <0.01 were considered statistically significant. All experiments were carried out in triplicate and repeated at least three times.

## Results

### TrkB is Upregulated in EC Tissues

**Figure 1:**
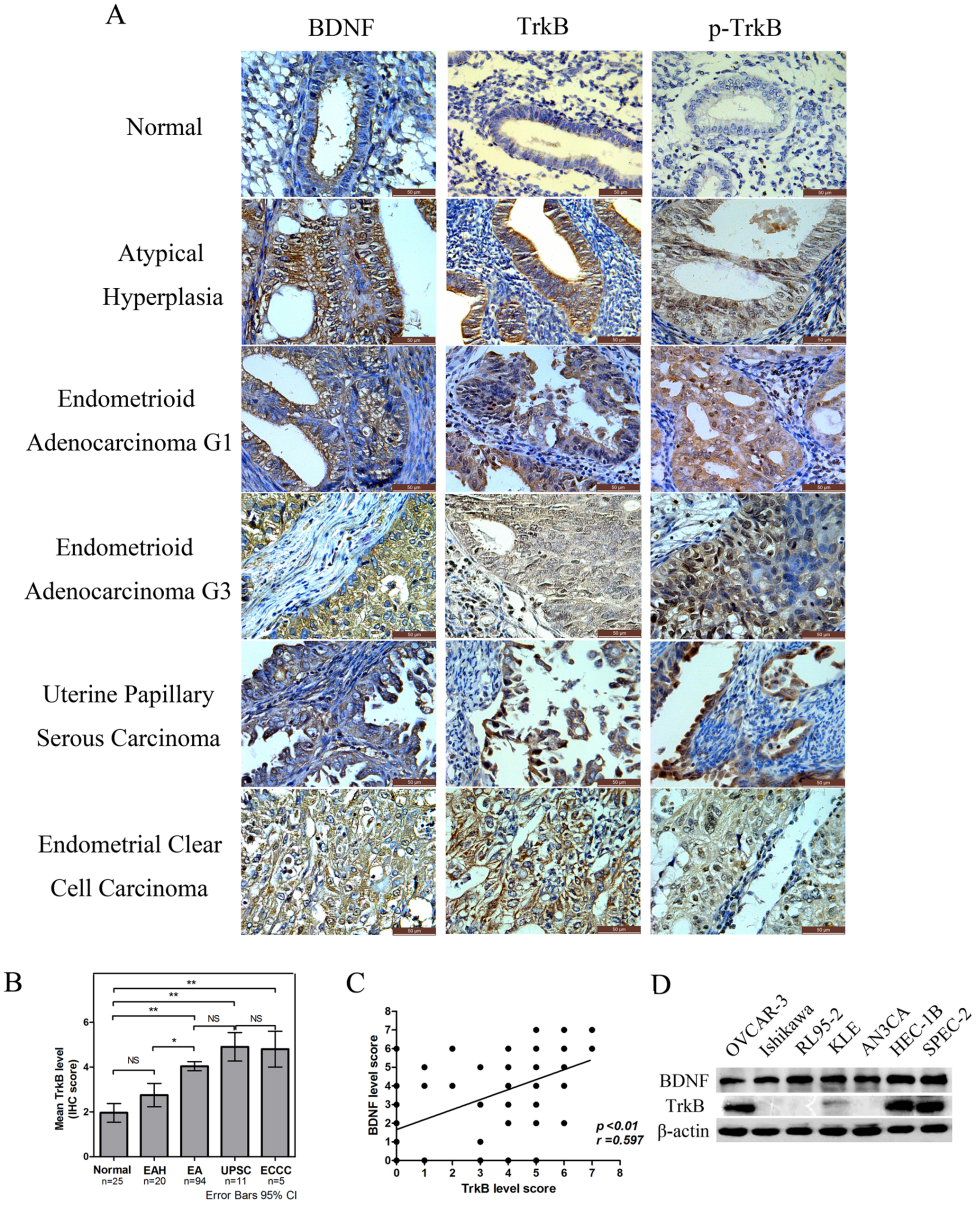
TrkB and BDNF expression in human EC and in EC cell lines. A. Immunohistochemical analysis of BDNF, TrkB and p-TrkB expression in normal endometrium, endometrial atypical hyperplasia (EAH), endometrioid adenocarcinoma (EA) G1/G3, uterine papillary serous carcinoma, and endometrial clear cell carcinoma (400×). No or weak expression of TrkB and BDNF was observed in normal endometrium, moderate expression in EAH, and strong cytoplasmic and cell membrane expression in the majority of tumors. B. Statistical summary of the immunostaining scores in normal endometrium, EAH, EA, UPSC and ECCC (*p<0.05, **p<0.01; NS, not significant). A score of ≥4 was considered positive for TrkB expression. C. A strong relationship between the TrkB and BDNF level scores in the 110 ECs was observed using a Spearman rank correlation coefficient (*r* = 0.597, *p*<0.01). D. Expression of TrkB and BDNF protein in six EC cell lines was measured by Western blotting. The human epithelial ovarian cancer cell lines OVCAR-3 was tested as a positive control. TrkB was highly expressed in HEC-1B and SPEC-2 cell lines and almost absent in Ishikawa, RL95-2, and AN3CA cell lines. All experiments were repeated at least three times.

Because increased TrkB expression has been associated with tumor progression of many human cancers, we sought to determine if this was also true for EC. The expression of TrkB protein in EC was analyzed by immunohistochemistry (IHC). TrkB protein was shown to be predominantly localized to the cytoplasm and cell membrane of endometrial epithelial cells. There was weak or no staining in normal endometrium, whereas moderate to strong TrkB immunostaining was observed in endometrial atypical hyperplasia and EC tissues (Figure 1A).

To account both for the stain intensity and the extent of staining, an IHC score (the sum of the intensity score and the extent score) was calculated. A total 110 cases of EC were histologically diagnosed as follows: Type I EC included endometrioid adenocarcinoma (n = 94), while type II EC consisted of uterine papillary serous carcinoma (UPSC) (n = 11) and endometrial clear cell carcinoma (ECCC) (n = 5). Among the different diagnostic groups, most of the normal endometrium were negative for TrkB (mean IHC score ≤2) and most of the EAH were weak or moderate for TrkB (mean IHC score <3), while almost all of the EC tissues were positive (mean IHC score >4) (Figure 1B). Protein expression of TrkB was significantly higher in EA (*p*<0.0001), UPSC (*p* = 0.0011) and ECCC (*p* = 0.0086) as compared to normal endometrium. These results are consistent with a role for TrkB in EC carcinogenesis. Moreover, of the 110 tumor samples analyzed, a strong correlation was noted (*r = *0.597, *p*<0.01, Figure 1C) between the expression of TrkB and its secreted ligand, BDNF, further supporting a potential role for this pathway.

We next explored the correlation of TrkB expression levels with clinicopathological parameters in EC. Significantly higher TrkB expression was found in carcinomas with lymph node metastasis (*p = *0.034, Table 1) and lymphovascular space involvement (*p = *0.045, Table 1). However, no association was found regarding patient age, FIGO staging, pathological grade, histological type, myometrial invasion, or expression of either the estrogen receptor (ER) or progesterone receptor (PR) (*p*>0.05, Table 1). These results suggest that TrkB expression correlates with both the occurrence of EC and risk-associated clinical features of the disease.

### TrkB Impacts Tumor Growth, Migration, and Invasion in vitro

qRT-PCR (Fig. S4) and Western blotting (Figure 1D) were performed to assess the expression of TrkB in endometrial cancer cell lines. The ovarian cancer cell line OVCAR-3 was tested as a positive control. Variable levels of TrkB and BDNF were detected across the EC cell lines. HEC-1B (high expression), Ishikawa (low expression), and RL95-2 (low expression) cell lines were chosen for further experimentation based on their differential expression of TrkB. To directly characterize the effects of TrkB on the oncogenic behavior of endometrial cancer cells, pLenti X1-shRNA-TrkB or pLenti X1-shRNA-nontarget (NT) were transfected into HEC-1B. Additionally, pLV.EX3d.P-TrkB or pLV.EX3d.P-empty vector (EV) were transfected into Ishikawa cells to establish stable cell lines with controlled levels of TrkB expression (Figs. S2 and S3). Stable transfection led to approximately 60% to 80% inhibition of TrkB expression in HEC-1B cells by shRNA-TrkB#1 and shRNA-TrkB#3 (Fig. S3). As a control for potential toxicity effects of lentiviral transduction, we found no difference between parental tumor cells and lentiviral transduced control cells by MTT assays (Figs. S2C and S3C).

**Figure 2:**
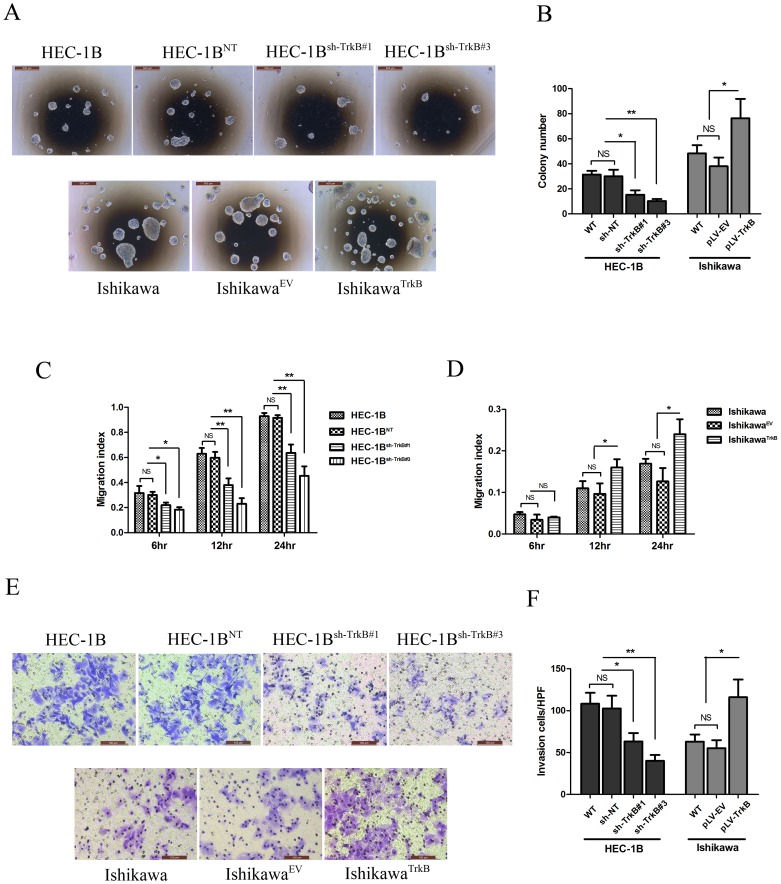
TrkB impacts tumor growth, migration, and invasion in vitro. A. Anchorage-independent growth in soft agar of EC cell lines with silenced or overexpressed TrkB (40×). Representative images are shown. B. Mean +/− SD of the number of soft agar colonies for EC cell lines with silenced or overexpressed TrkB. HEC-1B^sh−TrkB#1^ cells and HEC-1B^sh−TrkB#3^ showed decreased colony formation ability compared with HEC-1B^NT^ and wild type (*p<0.05, **p<0.01; NS, not significant), while Ishikawa^TrkB^ cells showed increased colony formation ability compared with Ishikawa^EV^ or parental Ishikawa cells (*p<0.05; NS, not significant). C. Cell migration was continuously tested by wound healing assay. HEC-1B^sh−TrkB#1^ and HEC-1B^sh−TrkB#3^ cells showed an obvious decrease in the migration index compared to HEC-1B or HEC-1B^NT^ cells during the time period (*p<0.05, **p<0.01; NS, not significant). D. Ishikawa^TrkB^ cells exhibited an obvious increase in migration index compared to Ishikawa^EV^ or wild type (*p<0.05; NS, not significant). E. Cell invasion was measured in transwell chambers (200×). Representative images are shown. F. Cells were counted with a microscope in five random high-powered fields. Invasion ability was significantly decreased in HEC-1B^sh−TrkB#1^ and HEC-1B^sh−TrkB#3^ cells (*p<0.05, **p<0.01; NS, not significant), and it was enhanced in Ishikawa^TrkB^ cells (*p<0.05; NS, not significant). **p<0.01, *p<0.05 considered significant. Bars show mean ± SD. All experiments were carried out in triplicate and repeated at least three times.

To evaluate the impact of TrkB inhibition on anchorage-independent growth of HEC-1B cells in soft agar, we compared the colony forming ability of the TrkB knockdown cells. Colony formation was inhibited in HEC-1B^sh−TrkB#1^ cells (*p*<0.05) and HEC-1B^sh−TrkB#3^ cells (*p*<0.01) compared with both HEC-1B^NT^ and wild type control cells (Figures 2A and 2B). Furthermore, the over-expression of TrkB significantly stimulated the growth of Ishikawa^TrkB^ cells compared to Ishikawa^EV^ or parental Ishikawa cells (Figures 2A and 2B).

To further determine the role of TrkB in cell migration, wound healing assays were performed. While HEC-1B and HEC-1B^NT^ cells could nearly close the wound after 24 h of incubation (migration index of close to 1), HEC-1B^sh−TrkB#1^ cells and HEC-1B^sh−TrkB#3^ cells were unable to do so after the same amount of time (*p*<0.01, Figure 2C). Furthermore, Ishikawa^TrkB^ cells exhibited an obvious increase in the migration index compared to Ishikawa^EV^ or wild type cells (*p*<0.05, Figure 2D). Invasion assays were also performed to verify the effect on motility of EC cells. Consistent with the results of the wound healing assay, after 48 h incubation, invasion ability was significantly decreased in HEC-1B^sh−TrkB#1^ cells (*p*<0.05) and in HEC-1B^sh−TrkB#3^ cells (*p*<0.01), but enhanced in Ishikawa^TrkB^ cells (*p*<0.05) (Figures 2E and 2F). These results suggest that TrkB may impact tumor metastatic activity in EC.

### TrkB Promotes Anoikis Resistance in Suspension Culture

**Figure 3:**
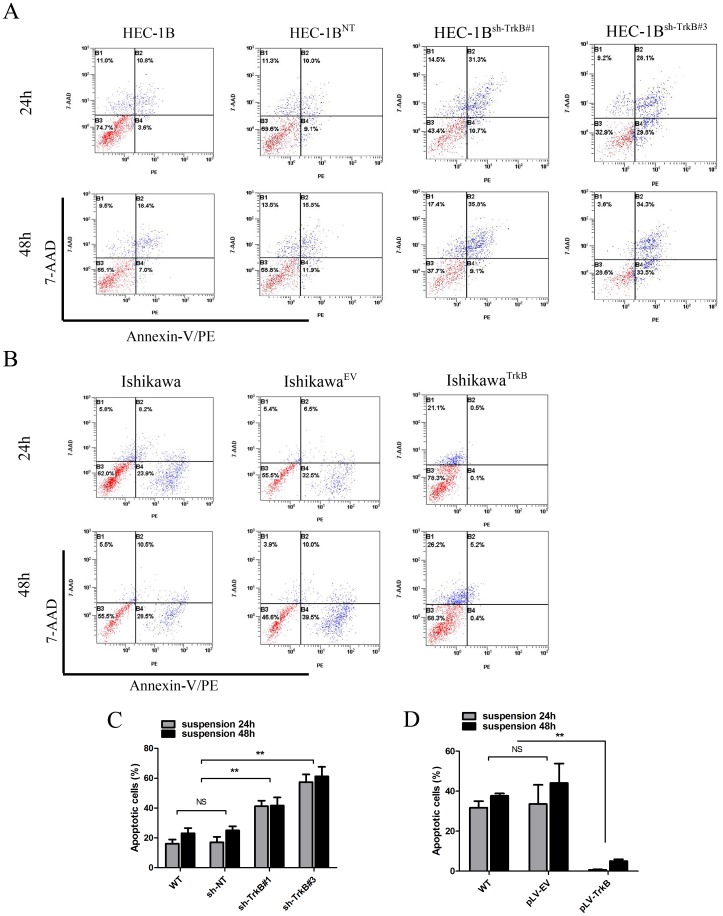
TrkB effects anoikis resistance in suspension culture. A. Representative images of flow cytometry analysis of apoptotic cells by Annexin V-PE/7-AAD staining in HEC-1B cell lines 24 h and 48 h after anchorage-independent culture. B. Representative images of flow cytometry analysis of apoptotic cells by Annexin V-PE/7-AAD staining in Ishikawa cell lines 24 h and 48 h after anchorage-independent culture. C and D. Flow cytometry results were plotted as the mean+SD of triplicate experiments at 24 h and 48 h after suspension culture. **p<0.01 considered significant; NS, not significant. All experiments were carried out in triplicate and repeated at least three times.

Because TrkB was recently identified as a potent anoikis suppressor [17], we tested its role in EC by inducing anoikis on anchorage-independent culture. Cells were analyzed by annexin V-PE/7-AAD staining and flow cytometry 24 h or 48 h after anoikis induction. After 24 h suspension, a greater percentage of HEC-1B^sh−TrkB#1^ cells (41.23±3.71%) and HEC-1B^sh−TrkB#3^ cells (57.43±5.12%) were apoptotic, compared to HEC-1B^NT^ cells (17.00±3.72%) or wild type cells (16.06±2.81%) (Figures 3A and 3C). Conversely, the number of apoptotic Ishikawa^TrkB^ cells (0.64±0.26%) was lower than the number of apoptotic Ishikawa^EV^ cells (33.57±9.58%) or apoptotic parental Ishikawa cells (31.70±3.32%) (Figures 3B and 3D). Similar results were observed for each cell type after 48 h suspension incubation, (*p*<0.01, Figures 3A–D), further verifying these findings. These data suggest that TrkB protects EC cells from anoikis in suspension culture.

### TrkB Induces EMT in EC Cell Lines

**Figure 4:**
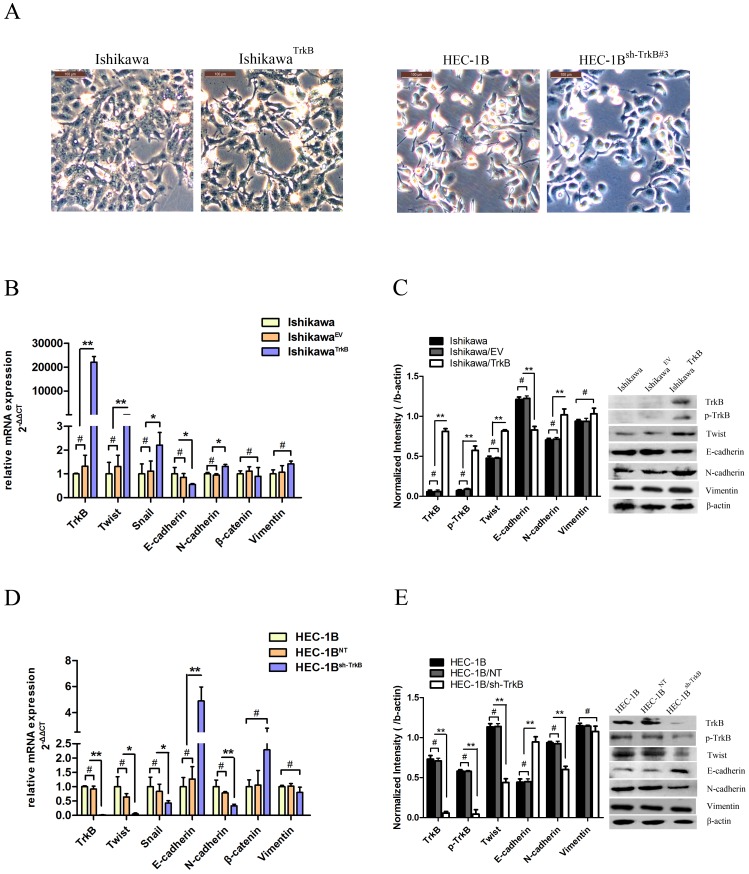
TrkB induce EMT in EC cell lines. A. Left: Mesenchymal morphology induced by over-expression of TrkB in Ishikawa cells (200×). Right: Epithelial morphology induced by suppression of TrkB in HEC-1B cells (200×). B. Messenger RNA (mRNA) levels of TrkB and EMT markers as analyzed by qRT-PCR in Ishikawa cells. β-actin was included as an internal control. (*p<0.05, **p<0.01; #, not significant). C. Protein levels of TrkB and EMT markers in Ishikawa cells as analyzed by immunoblotting (Right), and further quantified by densitometry of triplicate experiments (Left, **p<0.01; #, not significant). β-actin was included as an internal control. D. mRNA levels of TrkB and EMT markers as analyzed by qRT-PCR in HEC-1B cells (*p<0.05, **p<0.01; #, not significant). E. Protein levels of TrkB and EMT markers in HEC-1B cells as analyzed by immunoblotting (Right), and further quantified by densitometry of triplicate experiments (Left, **p<0.01; #, not significant). β-actin was included as an internal control. All experiments were carried out in triplicate and repeated at least three times.

We observed the cells under microscopy to determine the effects of TrkB on cellular morphology. Ishikawa^TrkB^ cells underwent a striking morphological transformation compared to parental cells. This change was characterized by a spindle-shaped morphology and the loss of cell-cell contacts (Figure 4A, left). Furthermore, the rounded and compact nature of the TrkB-knockdown HEC-1B^sh−TrkB#3^ cells reflected a transition from the spindle-like morphology of the parental cells to a more differentiated keratinocyte-like morphology, suggestive of a phenotypic transition from a mesenchymal morphology to an epithelial one (Figure 4A, right). To determine if this morphological transformation represented EMT (as was previously reported [25], [26]), we analyzed the levels of several characteristic epithelial and mesenchymal proteins by qRT-PCR and Western blotting. We observed decreased expression of E-cadherin and increased expression of N-cadherin in Ishikawa^TrkB^ cells (Figures 4B and 4C), while the reverse trend was observed for HEC-1B^sh−TrkB^ cells (Figures 4D and 4E). These results suggest that TrkB levels determine an EMT associated “cadherin switch” in both EC cell lines. Because the E boxes of the E-cadherin promoter are known to be repressed by the basic helix-loop-helix protein Twist [27], we assayed the effects of TrkB on the expression of this transcriptional repressor. Exogenous TrkB expression caused increased expression of Twist in Ishikawa^TrkB^ cells both at the mRNA (Figure 4B) and protein levels (Figure 4C), while conversely, suppressing of TrkB decreased the expression of Twist in HEC-1B^sh−TrkB^ cells (Figure 4D and E).

### Twist is Required for TrkB-induced EMT-like Transformation and Anoikis Suppression

**Figure 5:**
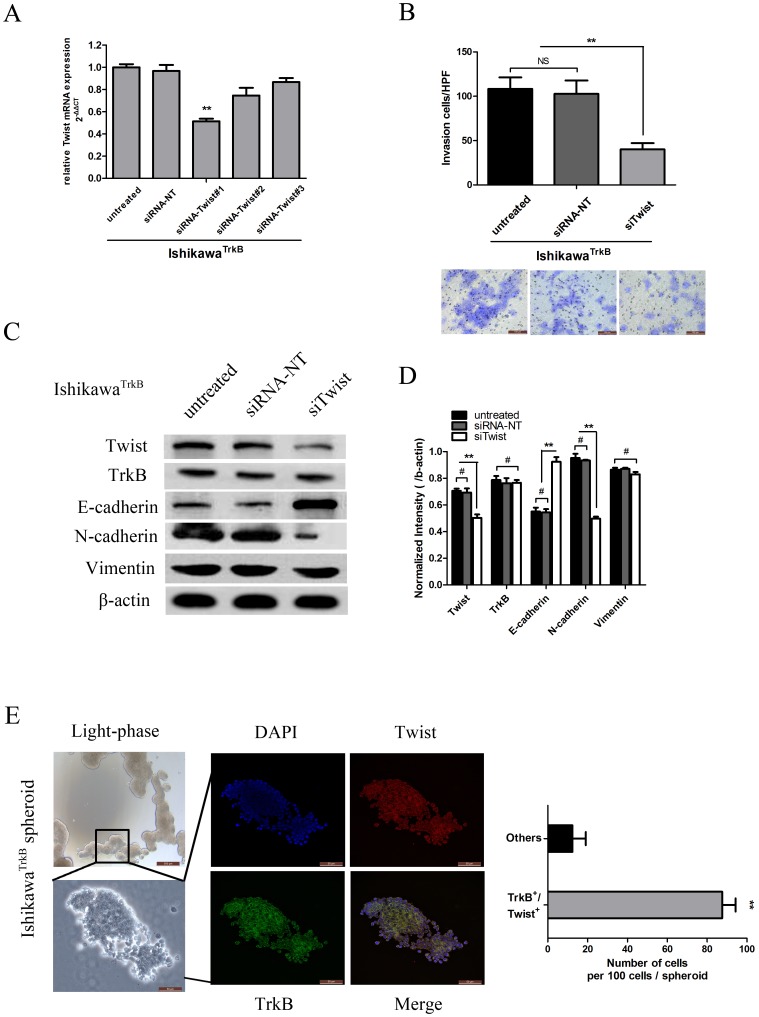
Twist is required for TrkB-induced EMT-like transformation and anoikis suppression. A: Quantification was performed to determine the relative change in Twist mRNA expression following transfection of Ishikawa^TrkB^ cells with control (siRNA-NT) or siRNA-Twist oligonucleotide duplexes. SiRNA-Twist#1 was chosen for further study (**p<0.01). B. Invasion ability was significantly decreased in Ishikawa^TrkB^ cells transfected with siRNA-Twist compared to untreated cells or cells transfected with siRNA-NT as assessed by transwell assay(**p<0.01; NS, not significant), Bars show mean ± SD. (Below, 200×). C. Protein levels of Twist, TrkB and EMT markers as analyzed by immunoblotting in Ishikawa^TrkB^ cells (Left), and further quantified by densitometry of triplicate blots (Right, **p<0.01; #, not significant). β-actin was included as an internal control. E. Surviving Ishikawa^TrkB^ cell spheroids in suspension culture (Left, upper: 40×, below: 400×). After adhering for 4 h on glass coverslips, spheres were immunostained with antibodies against Twist (red) and TrkB (green). In the merged images (bottom right), these spheres contain double-positive cells (400×). Quantification of these cell aggregates formed by surviving Ishikawa^TrkB^ cells showed a higher number of TrkB^+^/Twist^+^ cells than non-expressing cells (Right panel). The data are reported as mean ± SD (**p<0.01). All experiments were carried out in triplicate and repeated at least three times.

To directly address whether the effects of TrkB in promoting EC cell invasiveness are attributed to its activation of Twist, a rescue experiment was performed. We transfected Ishikawa^TrkB^ cells with three different Twist siRNA oligonucleotide duplexes or with control non-targeting siRNA (siRNA-NT), and determined that siRNA-Twist#1 significantly reduced Twist RNA levels as compared to siRNA-NT or untreated (*p*<0.01, Figure 5A). Knockdown of Twist by siRNA-Twist#1 inhibited the invasion ability of Ishikawa^TrkB^ cells as assessed by transwell invasion analysis (*p*<0.01, Figure 5B) and promoted the “cadherin switch” from N-cadherin to E-cadherin (Figure 5C and D). These results suggest that the effects of TrkB in mediating an EMT-like transformation of EC cells are mediated by Twist.

To determine if the EMT phenotype associated with TrkB expression is essential for anoikis resistance, we induced anoikis in Ishikawa^TrkB^ cells by culturing cells on Poly-HEMA treated plates. The cells gradually formed increased numbers of cell spheroids (Figure 5E, left). As a key EMT regulator, Twist was found to be co-expressed with TrkB in these cell spheroids (Figure 5E, middle). Quantification of these cell aggregates demonstrated that the majority of the surviving Ishikawa^TrkB^ cell population was both TrkB^+^ and Twist^+^ (Figure 5E, right). The coordinate expression of these two proteins is supported by comparative analyses of human EC (Fig. S5). These results imply that Twist expression is coincident with TrkB expression for cells following a path towards EC cell invasion and EMT-like transformation, further suggesting that Twist is required for TrkB-induced EMT-like transformation and anoikis resistance.

### Activation of TrkB by BDNF Promotes Carcinogenesis in EC

**Figure 6:**
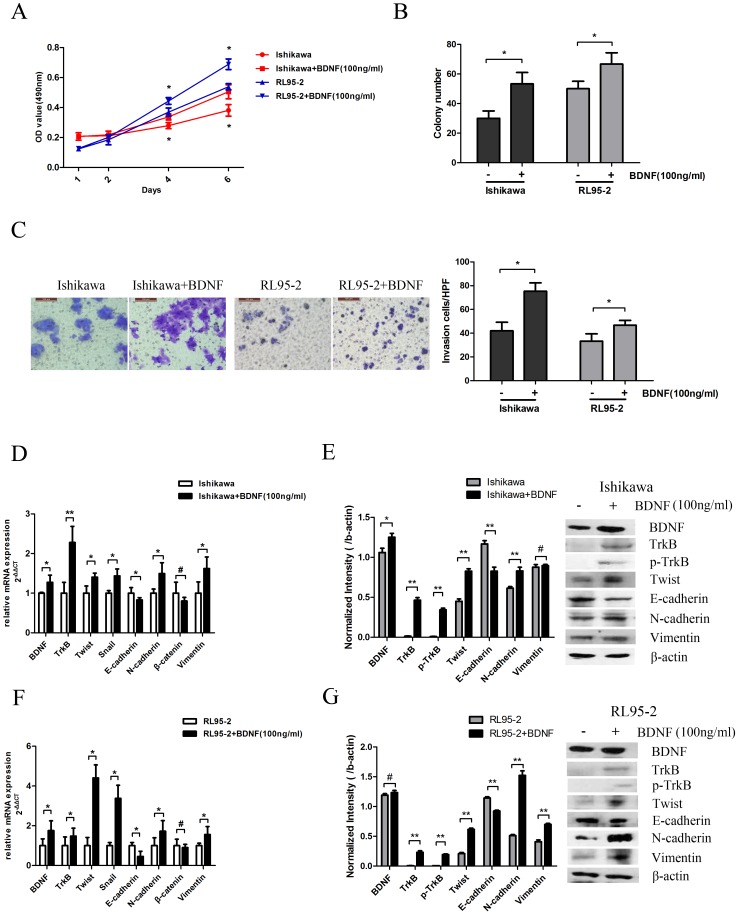
Activation of TrkB by BDNF promotes carcinogenesis in EC. A. After BDNF (100 ng/ml) treatment for 24 h, the effects on the proliferation of Ishikawa and RL95-2 cells were determined by MTT assay (*p<0.05). B. BDNF(100 ng/ml) treatment for 24 h promoted tumor growth of Ishikawa and RL95-2 cells as assessed by colony formation in soft agar (*p<0.05). C. Left: Cell invasion was detected in transwell chambers with or without 24 h BDNF (100 ng/ml) treatment (200×). Representative images are shown. Right: Graphical representation of the fold change in the number of invasive cells in Ishikawa and RL95-2 cells following BDNF treatment. *p<0.05 is considered significant. Bars show mean ± SD. D. mRNA levels of BDNF, TrkB, and EMT markers as analyzed in Ishikawa cells by qRT-PCR 24 h after BDNF treatment. β-actin was included as an internal control (*p<0.05, **p<0.01; #, not significant). E. Protein levels of BDNF, TrkB, and EMT markers as analyzed in Ishikawa cells by Western blotting 48 h after BDNF treatment (Right), and further quantification by densitometry of triplicates (Left, *p<0.05, **p<0.01; #, not significant). β-actin was included as an internal control. F. mRNA levels of BDNF, TrkB, and EMT markers in RL95-2 cells as analyzed by qRT-PCR 24 h after BDNF treatment. β-actin was included as an internal control (*p<0.05; #, not significant). G. Protein levels of BDNF, TrkB, and EMT markers as analyzed in RL95-2 cells followed by Western blotting 48 h after BDNF treatment (Right). The results were further quantified by densitometry of triplicates (Left, **p<0.01; #, not significant). β-actin was included as an internal control. All experiments were carried out in triplicate and repeated at least three times.

Studies in neuroblastoma cell lines have shown that BDNF stimulation induces TrkB-mediated cell invasion [28]. To specify a role for BDNF in TrkB signaling in EC, we also examined whether BDNF-TrkB signaling affects cellular proliferation, motility, and invasion in EC cells. Activation by BDNF promoted significant upregulation of tumor cell growth (Figure 6A and 6B) and motility (Figure 6C) of both Ishikawa and RL95-2 cell lines. This was accompanied by increased expression of TrkB and p-TrkB (Figures 6E and 6G). Furthermore, decreased expression of E-cadherin and increased expression of Twist, N-cadherin, and Vimentin was detected by qRT-PCR and Western blotting in both Ishikawa cells (Figure. 6D and 6E) and RL95-2 cells (Figure. 6F and 6G). Collectively, these results suggest that the migratory and invasive properties of EC may be mediated by a BDNF-TrkB signaling cascade.

### Suppression of TrkB Abrogates Tumor Peritoneal Dissemination in vivo

**Figure 7:**
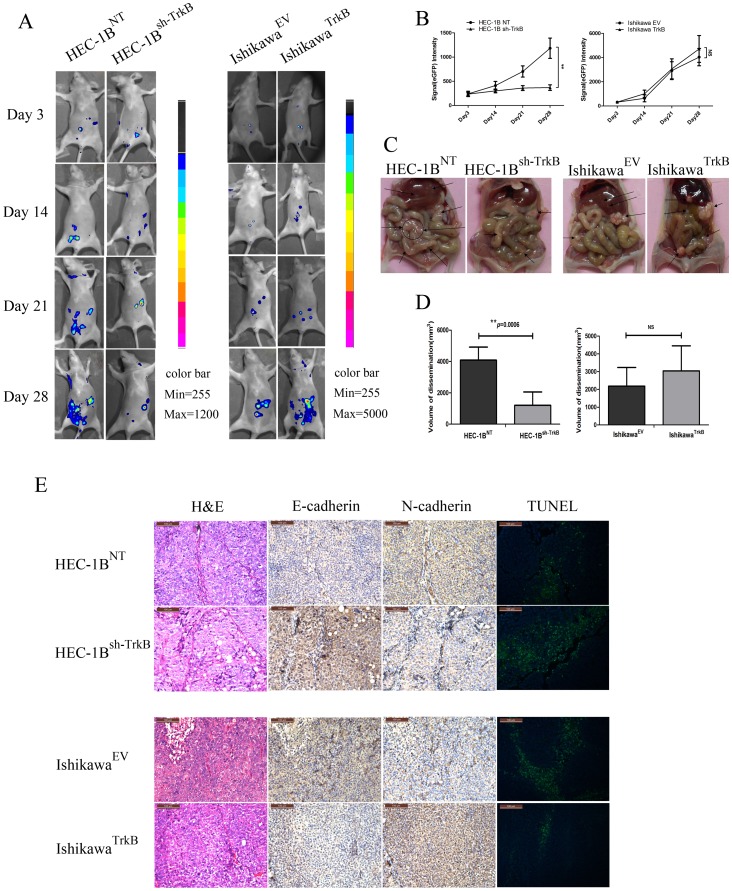
Suppression of TrkB abrogates tumor peritoneal dissemination in vivo. A. Cells were injected i.p. into mice. Abdominal colonization was measured using fluorescence images at 3, 14, 21 and 28 days after injection. The color bar shows photon intensity. B. A bar graph of the average fluorescent signal intensities from 5 mice at day 3, 14, 21 and 28 after injection is shown (**p<0.01; NS, not significant). C. Pathologic characteristics of dissemination in TrkB knockdown and TrkB over-expression xenografts. A significantly lower tumor burden was observed in mice from the HEC-1B^sh−TrkB^ group compared with those from the HEC-1B^NT^ group as indicated by black arrows. D. Left: Average volume of tumor dissemination in the HEC-1B^sh−TrkB^ group was lower than that in the HEC-1B^NT^ group (n = 5, **p<0.01). Right: There is no significant difference in the average volume of tumor dissemination between the Ishikawa^TrkB^ group and the Ishikawa^EV^ group (n = 5, NS, not significant). E. H&E staining, IHC, and TUNEL analysis of tumors sections. Upper: Alterations in N-cadherin and E-cadherin were confirmed by IHC (200×). Apoptotic cells (green, 200×) were increased in the HEC-1B^sh−TrkB^ group. Lower: Alterations in E-cadherin and N-cadherin were confirmed by IHC, and apoptotic cells were decreased in Ishikawa^TrkB^ group mice. All experiments were repeated at least three times.

To test the hypothesis that TrkB potentiates EC metastasis, peritoneal disseminated tumor growth assays in nude mice were performed. HEC-1B^NT^ or HEC-1B^sh−TrkB^ and Ishikawa^EV^ or Ishikawa^TrkB^ were injected intraperitoneally (i.p.) into nude mice, which were then serially observed by fluorescence imaging for 4 weeks (Figure 7A). Two weeks after injection, the eGFP fluorescence intensity in the abdominal regions of mice from the HEC-1B^NT^ group was higher than those from the HEC-1B^sh−TrkB^ group, and the higher level was maintained during the entire observation period (Figure 7B). Pathologic anatomy analysis of the tumor volume of dissemination revealed a significant abrogation of tumor growth in the HEC-1B^sh−TrkB^ group compared with the HEC-1B^NT^ group (*p*<0.001, Figures 7C and 7D, left). Though there was a trend towards increased tumor volume of dissemination in the Ishikawa^TrkB^ group, no significant differences between the Ishikawa^TrkB^ group and the Ishikawa^EV^ group were observed (*p*>0.05, NS, Figures 7C and 7D, right), suggesting that the extent of the in vivo effect of TrkB may be cell-dependent.

To verify these results and to further examine the pathways of activation by TrkB in vivo, we performed immunohistochemical staining of paraffin-embedded sections. Upregulation of E-cadherin was observed in HEC-1B^sh−TrkB^ tumors, recapitulating the mesenchymal-to-epithelial transition in vivo (Figure 7E). Conversely, apoptosis was increased in the HEC-1B^sh−TrkB^ tumors compared with the HEC-1B^NT^ tumors as determined by TUNEL assay. Once again, for the Ishikawa cells, a trend was observed that the TrkB-expressing tumors expressed less E-cadherin and had less apoptosis than the control tumors; however results were not as dramatic as for the HEC-1B cells. Taken together, these results suggest an important role for TrkB in regulating tumor viability and anoikis resistance in EC.

## Discussion

Tumor dissemination and metastasis are the leading causes of death in endometrial cancer. It has been long recognized that TrkB is oncogenic in tumors of neurogenic origin, such as neuroblastoma [29]. However, recent work has shown that its oncogenic role in other tumors, including its ability to regulate EMT, is quite distinct from its role in neurogenic diseases [30]. Here, we show that TrkB and its stimulatory ligand, BDNF, are highly expressed in human EC. Specific knockdown or over-expression of TrkB in endometrial cancer cell lines led to changes in tumor growth and metastatic potential in vitro and in vivo. Our results suggest that TrkB expression may regulate EC-associated metastatic potential.

Although several studies have investigated the expression of TrkB in solid cancers [6]–[8], this is the first study to address the function of TrkB in human EC. Anger et al. [31] showed that TrkB protein is expressed in human endometrium, and its expression may be greater in women with endometriosis. This finding implies that human endometrium has ectopic growth traits. We found that the normal endometrium produces weak levels of TrkB. but that TrkB was extensively detected in EC. A correlation between TrkB and BDNF expression was also found in EC, which is in accordance with reports of other tumors [32], [33]. Previous studies demonstrate that TrkB is an independent prognostic factor for ovarian and gastric cancers [23], [34]. Consistently, our results revealed that high TrkB levels are associated with lymph node metastasis and lymphovascular space involvement, which are associated with high risk factors in EC patients. Expression of TrkB has also been associated with poor tumor pathological grade or advanced tumor stage in several solid tumor entities including ovarian cancer, lung cancer and colorectal carcinoma [23], [35], [36], however, our exploratory clinical study could not confirm this association statistically in EC. Nevertheless, even with our limited patient numbers, a trend is observed that the expression of TrkB in EC pathological grade G3 (7/9, 77.8%) is slightly higher than that in G1 (33/45, 73.3%) and G2 (31/40, 77.5%) (Table 1). Furthermore, though no statistical correlation was observed in TrkB expression for the 110 patient samples categorized by histological type, we demonstrated that the mean TrkB level in UPSC and ECCC were higher than in endometrioid adenocarcinoma (Figure 1B). Thus, though TrkB expression is clearly upregulated in EC, correlates with EC-associated risk factors and has a clear role in metastatic potential, future studies with larger numbers of patients will be necessary to precisely define the correlation of TrkB expression with the EC grade or histological type. Though these results are not sufficient to definitively designate TrkB as a prognostic factor for EC, our expression data combined with our functional data suggest that TrkB might serve as a target for anticancer therapy.

We show that the rate of anoikis in HEC-1B cells is increased by TrkB knockdown. Conversely, Ishikawa cells over-expressing TrkB showed decreased rates of anoikis. Anoikis is defined as apoptosis resulting from loss of cell–matrix interactions and represents a physiological barrier to metastasis [37]. These data, therefore, suggest that TrkB is an important regulator of a characteristic metastatic process in EC cells. Although the underlying mechanisms governing anoikis resistance in cancer cells are not fully understood, members of the Bcl-2 family of proteins have been implicated in susceptibility to anoikis [38]. In non-transformed intestinal cells, anoikis was associated with detachment-induced down-regulation of Bcl-XL, and it did not involve activation of caspase-9 [39]. In addition, Activation of the phosphoinositide 3-kinase (PI3K)/Akt pathway has a widespread role in cell survival signaling in general, and anoikis is no exception. It was reported that PI3K is an important downstream target of the cancer cell survival pathway in the resistance of tumor cells to anoikis [40]. Additional studies are necessary to identify whether these factors are involved in anoikis resistance by TrkB in EC.

However, our results also identify a role for Twist in the process of anoikis resistance. Twist is known to be a key regulator of EMT, a process by which tumor epithelial cells are re-programmed so that they no longer require attachments for their survival and thus achieve anoikis resistance [41]. Twist plays a critical role in breast cancer metastasis [42]. Furthermore, high Twist expression in infiltrative EC affects patient survival [22], though the mechanism of EMT in EC remains unclear. Results from our study support high levels of Twist in EC, which are significantly associated with TrkB expression (Fig. S5). Furthermore, we show that levels of Twist expression are modulated by TrkB and are required for TrkB-induced EMT transformation and tumorigenesis in vitro (Figures 4–6). Notably, in anchorage-independent culture, we found that both TrkB and Twist were expressed in survival cell spheroids, and many EMT-related genes were differentially expressed compared to adherent cells (Fig. S6), which are hallmarks of the oncogenic EMT being required for anoikis resistance.

Peritoneal dissemination of EC cells is a multistep process consisting of invasion into the serosa from the uterus, detachment from the primary site, movement into the peritoneal cavity, attachment to the distant peritoneum, invasion into the subperitoneal space, and proliferation [43]. From our in vivo experiments, we discovered that the TrkB knockdown significantly inhibited the establishment of intraperitoneal disseminated tumors. A constitutive “cadherin switch” and apoptosis shift were also detected in paraffin-embedded sections of mouse tumors, which are consistent with the in vitro results.

Mechanistically, Trks are receptor tyrosine kinases activated by neurotrophins and other growth factors. TrkA, TrkB and TrkC are the preferred receptors for the neurotrophins NGF, BDNF and neurotrophin-3 (NT-3), respectively [44]. When stimulated by BDNF ligand, TrkB induces the activation of various downstream signaling pathways including Akt, Src, or MAPK resulting in cell proliferation, and apoptosis resistance in models of human cancer [12], [45]. In the present study, we found that both BDNF and TrkB are over-expressed in human endometrial carcinoma specimens. In vitro experiments verified that BDNF enhances cell proliferation and survival. In addition, the migratory and invasive properties of EC are mediated by a BDNF-TrkB signaling cascade in Ishikawa and RL95-2 cells. After the discovery of the involvement of the BDNF/TrkB cascade in cancer biology, it is appreciated that the inhibition of TrkB activity might be beneficial in a clinical oncology setting [46]. Previous studies have demonstrated a key role for BDNF-TrkB signaling in modulating the response to cytotoxic agents, and modulation of TrkB expression enhanced the sensitivity of cells to cis-Diamminedichloroplatinum Cisplatin (CDDP) in head and neck squamous cell carcinoma [16]. Recently, It was reported Trk inhibitor K252a inhibited cell growth and induced apoptotic cell death in uterine leiomyosarcoma [47].Therefore, further studies are needed to explore the development of small-molecule compounds that act as TrkB antagonists, or monoclonal antibodies against either BDNF or TrkB, as promising novel therapies for the treatment of endometrial carcinoma. Our findings provide new insights into biological mechanisms of EC and establish TrkB as a potential target for future therapies for this disease.

## Supporting Information

Figure S1 **Verification of the specificity of the TrkB and p-TrkB IHC using paraffin embedded HEC-1B^NT^ and HEC-1B^sh^** ^−**TrkB**^ **cell pellets as a control.** H&E and IHC analysis of TrkB and p-TrkB expression in paraffin embedded HEC-1B^NT^ and HEC-1B^sh−TrkB^ cell pellets (400×). No or weak staining of TrkB and p-TrkB was observed in HEC-1B^sh−TrkB^ cells, and strong cytoplasmic and cell membrane staining of TrkB and p-TrkB in HEC-1B^NT^ cells. All experiments were repeated at least three times.
(TIF)

Figure S2 **Verification of stable transfection efficiency induced by over-expression of TrkB.** A. mRNA (top) and protein (bottom) levels of TrkB after stable transfection of Ishikawa cells with empty vector or TrkB vector. B. Cellular morphology of stably transfected cells (magnification 100×) under light (top) or fluorescence (bottom) microscopy. C. The effects on the proliferation of Ishikawa and Ishikawa^EV^ cells was determined by MTT assay to ensure that cells tolerate the lentiviral transduction without significant cytotoxicity (NS, not significant). All experiments were carried out in triplicate and repeated at least three times.
(TIF)

Figure S3 **Verification of stable transfection efficiency and targeted reduction of TrkB.** A. Left: mRNA (top) and protein (bottom) levels of TrkB after stable transfection of HEC-1B cells with vectors targeting an irrelevant sequence (shRNA-NT) or TrkB (shRNA-TrkB). Right: Quantification was performed to determine the relative change in TrkB mRNA expression among the various constructs. Vector shRNA-TrkB#3 and shRNA-TrkB#1 were selected for further study (**p<0.01). B. Cellular morphology of stably transfected cells (magnification 100×) under light (top) or fluorescence (bottom) microscopy. C. The effects on the proliferation of HEC-1B and HEC-1B^NT^ cells was determined by MTT assay to ensure that cells tolerate the lentiviral knockdown without significant cytotoxicity (NS, not significant). All experiments were carried out in triplicate and repeated at least three times.
(TIF)

Figure S4 **TrkB and BDNF expression in endometrial cancer cell lines.** qRT-PCR was performed using sequence-specific primers to detect BDNF (A) and TrkB (B) mRNA levels across different EC cell lines. β-actin was included as an internal control (error bars represent SD). The human epithelial ovarian cancer cell line OVCAR-3 was used as a positive control. All experiments were carried out in triplicate and repeated at least three times.
(TIF)

Figure S5 **High Twist expression in human EC is associated with TrkB expression.** A. Histogram summarizing the immunostaining scores of Twist in normal endometrium, EAH, and EC (**p<0.01; NS, not significant). The level of Twist was higher in tumor than in normal endometrium. B. The relationship between TrkB and Twist level score in 110 ECs was verified using a Spearman rank correlation coefficient (*r* = 0.418, *p*<0.01). All experiments were repeated at least three times.
(TIF)

Figure S6 **EMT markers are altered in anchorage-independent culture.** A. Two kinds of EC cell aggregates in anchorage-independent culture (magnification, 40×). B. EMT markers in suspension and adherent cells were determined by qRT-PCR and assayed with the primers as mentioned. β-actin was included as a loading control. *p<0.05, **p<0.01; #, not significant. These experiments were repeated three times with similar results.
(TIF)
