# Supplementary figures and images for: Upregulation of TrkB Promotes Epithelial-Mesenchymal Transition and Anoikis Resistance in Endometrial Carcinoma
Source: PLoS One. 2013 Jul 30;8(7):e70616. doi: 10.1371/journal.pone.0070616 (PMC3728299; doi:10.1371/journal.pone.0070616)

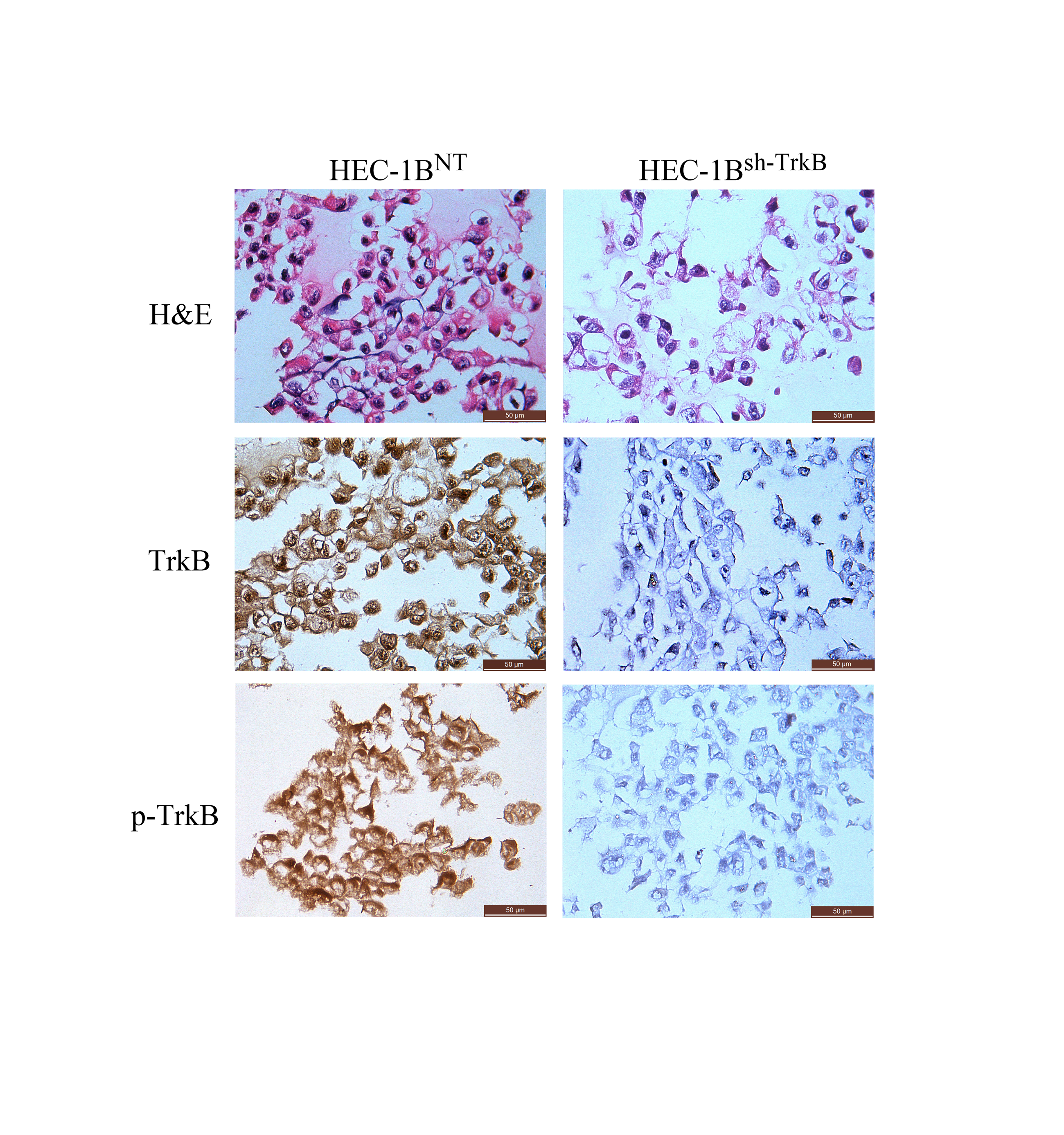

Supplement: Figure S1 — Verification of the specificity of the TrkB and p-TrkB IHC using paraffin embedded HEC-1BNT and HEC-1Bsh −TrkB cell pellets as a control. H&E and IHC analysis of TrkB and p-TrkB expression in paraffin embedded HEC-1BNT and HEC-1Bsh−TrkB cell pellets (400×). No or weak staining of TrkB and p-TrkB was observed in HEC-1Bsh−TrkB cells, and strong cytoplasmic and cell membrane staining of TrkB and p-TrkB in HEC-1BNT cells. All experiments were repeated at least three times. (TIF) [file pone.0070616.s001.tif]

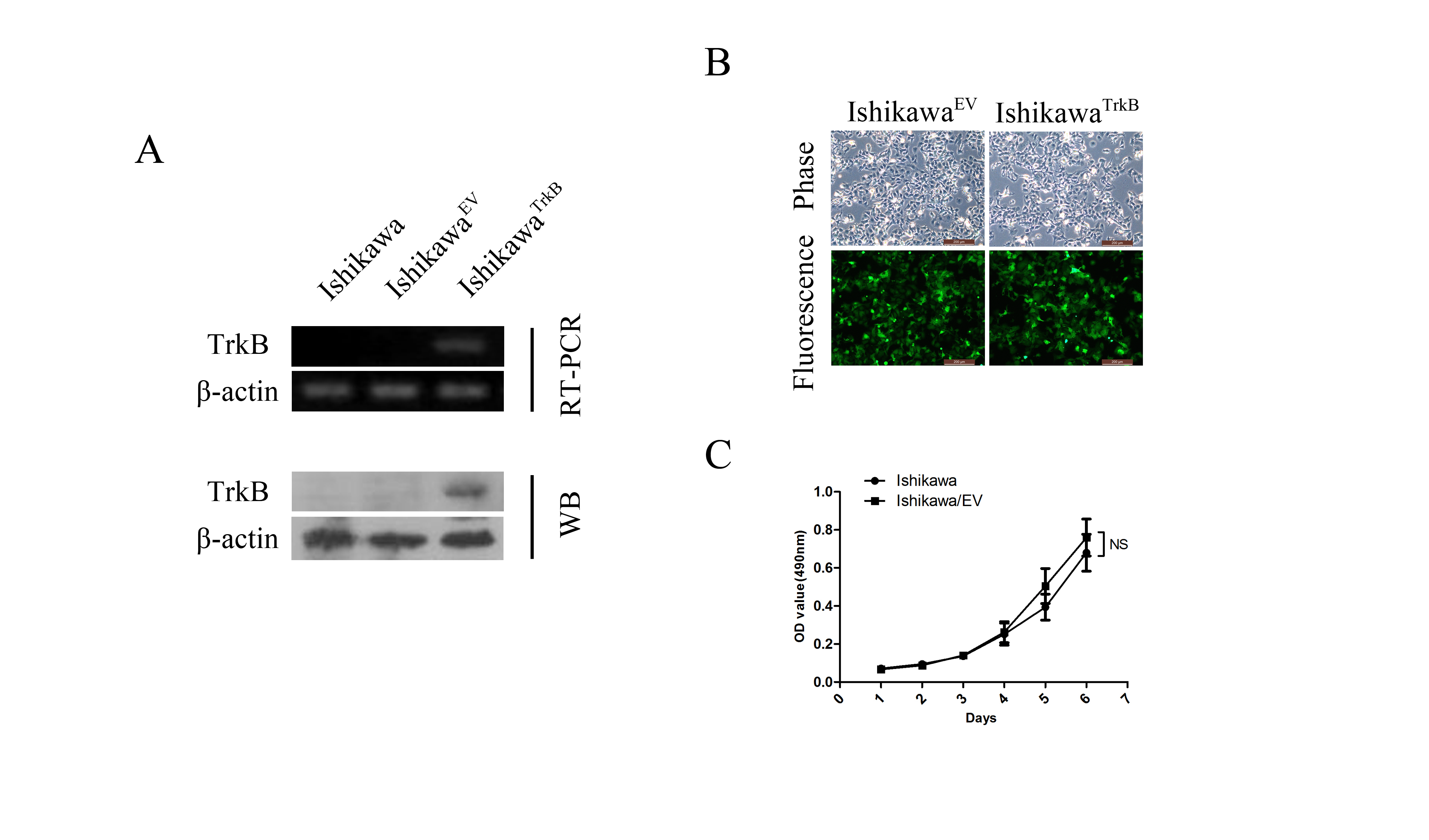

Supplement: Figure S2 — Verification of stable transfection efficiency induced by over-expression of TrkB. A. mRNA (top) and protein (bottom) levels of TrkB after stable transfection of Ishikawa cells with empty vector or TrkB vector. B. Cellular morphology of stably transfected cells (magnification 100×) under light (top) or fluorescence (bottom) microscopy. C. The effects on the proliferation of Ishikawa and IshikawaEV cells was determined by MTT assay to ensure that cells tolerate the lentiviral transduction without significant cytotoxicity (NS, not significant). All experiments were carried out in triplicate and repeated at least three times. (TIF) [file pone.0070616.s002.tif]

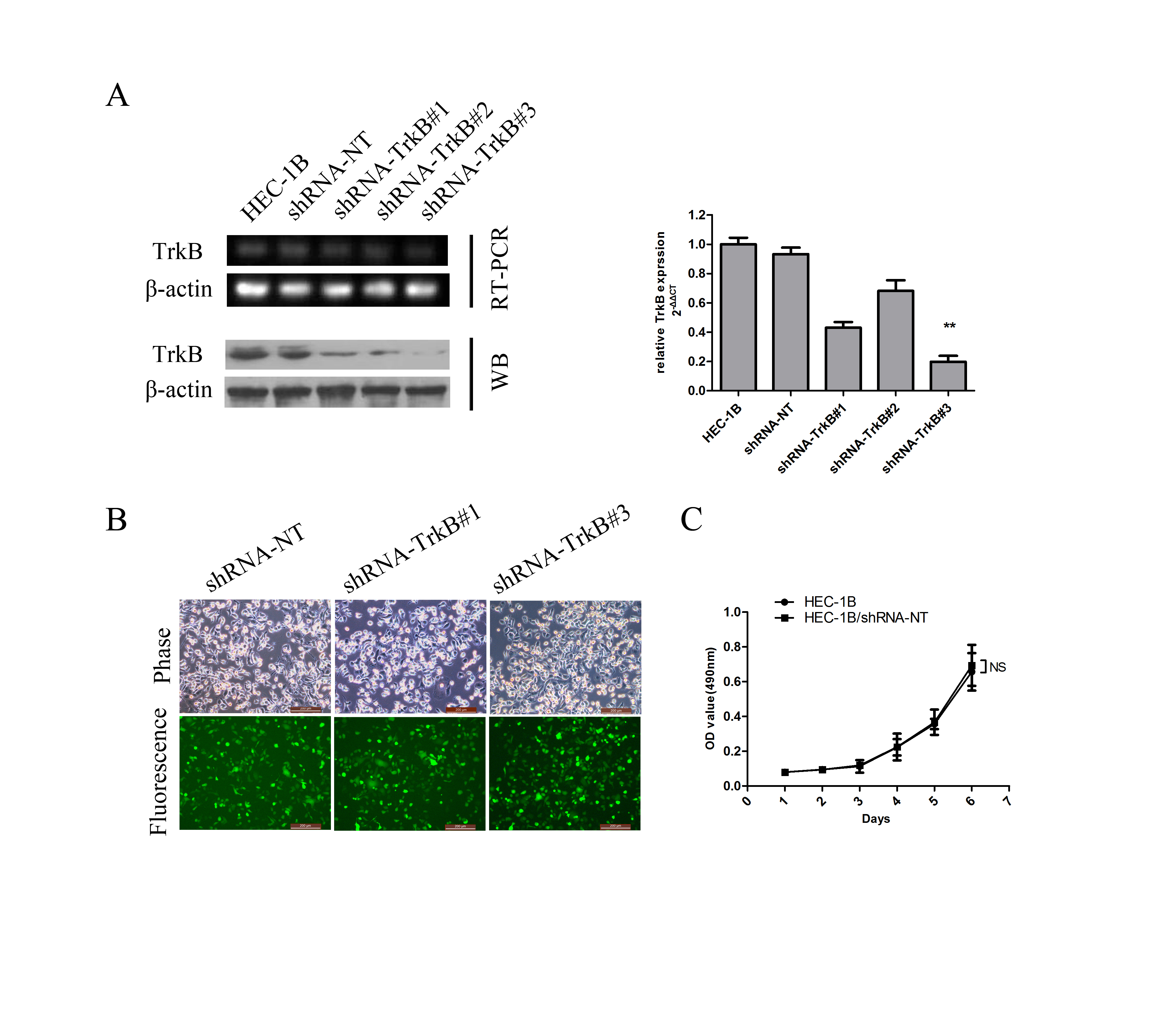

Supplement: Figure S3 — Verification of stable transfection efficiency and targeted reduction of TrkB. A. Left: mRNA (top) and protein (bottom) levels of TrkB after stable transfection of HEC-1B cells with vectors targeting an irrelevant sequence (shRNA-NT) or TrkB (shRNA-TrkB). Right: Quantification was performed to determine the relative change in TrkB mRNA expression among the various constructs. Vector shRNA-TrkB#3 and shRNA-TrkB#1 were selected for further study (**p<0.01). B. Cellular morphology of stably transfected cells (magnification 100×) under light (top) or fluorescence (bottom) microscopy. C. The effects on the proliferation of HEC-1B and HEC-1BNT cells was determined by MTT assay to ensure that cells tolerate the lentiviral knockdown without significant cytotoxicity (NS, not significant). All experiments were carried out in triplicate and repeated at least three times. (TIF) [file pone.0070616.s003.tif]

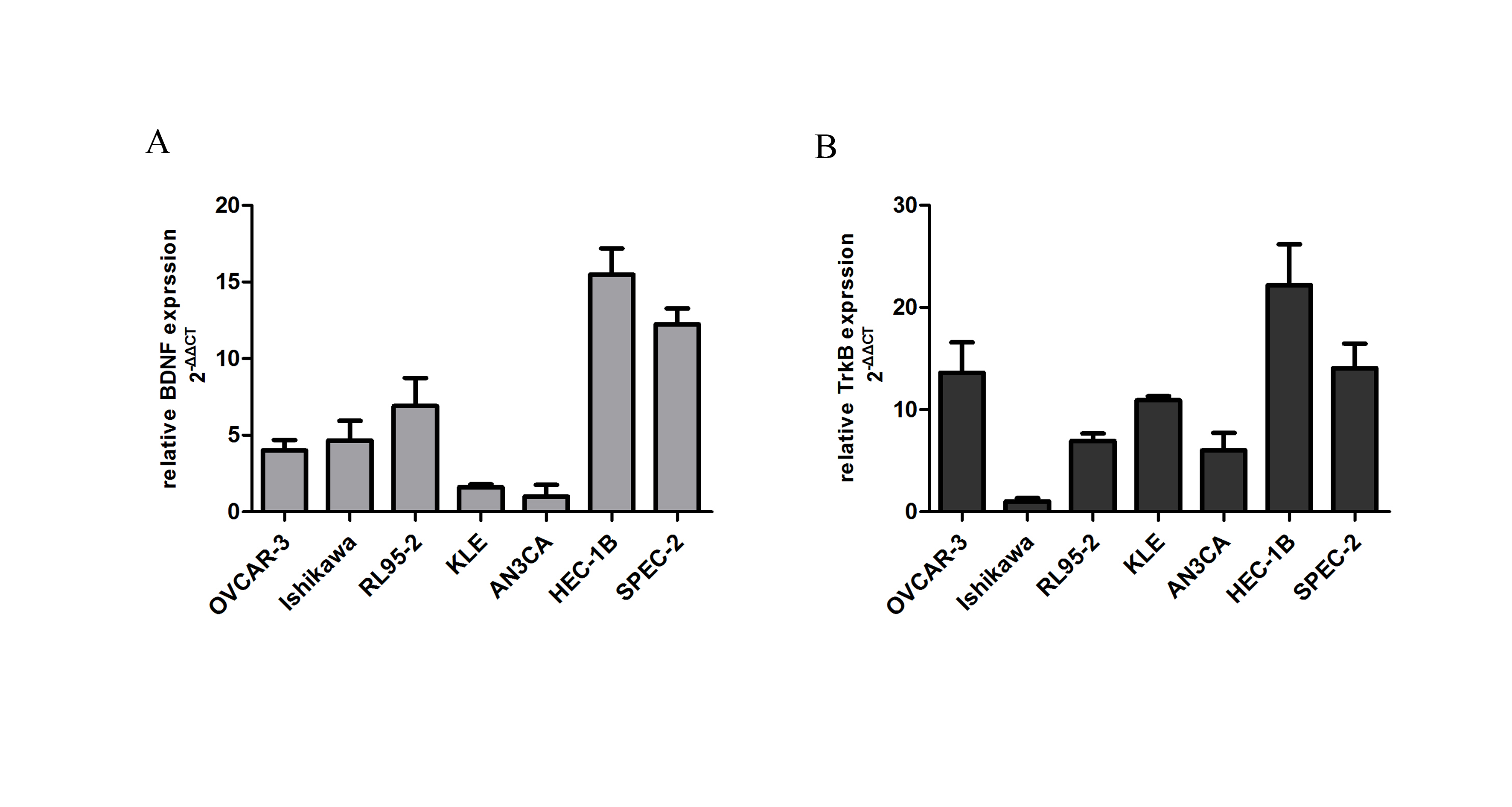

Supplement: Figure S4 — TrkB and BDNF expression in endometrial cancer cell lines. qRT-PCR was performed using sequence-specific primers to detect BDNF (A) and TrkB (B) mRNA levels across different EC cell lines. β-actin was included as an internal control (error bars represent SD). The human epithelial ovarian cancer cell line OVCAR-3 was used as a positive control. All experiments were carried out in triplicate and repeated at least three times. (TIF) [file pone.0070616.s004.tif]

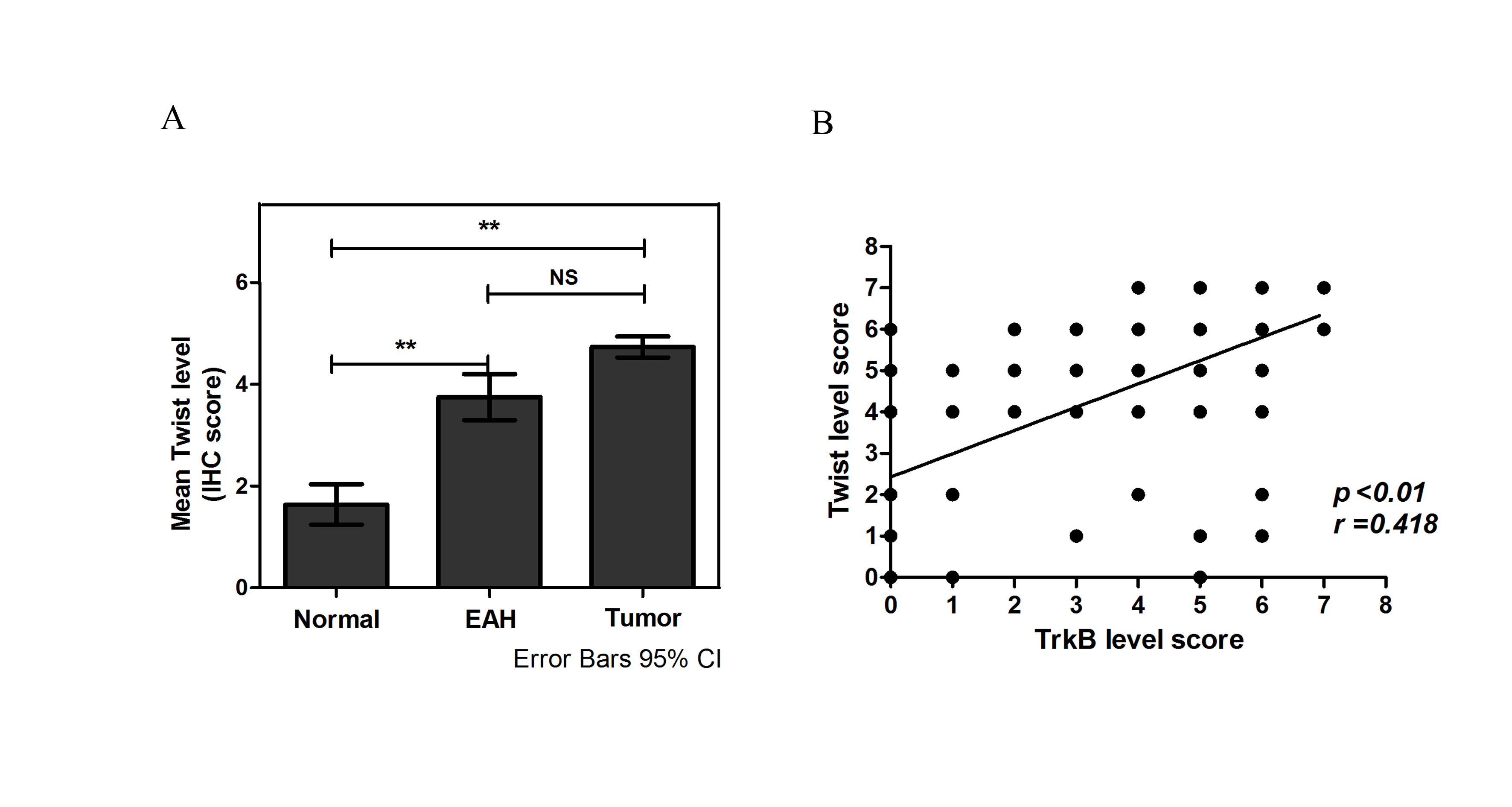

Supplement: Figure S5 — High Twist expression in human EC is associated with TrkB expression. A. Histogram summarizing the immunostaining scores of Twist in normal endometrium, EAH, and EC (**p<0.01; NS, not significant). The level of Twist was higher in tumor than in normal endometrium. B. The relationship between TrkB and Twist level score in 110 ECs was verified using a Spearman rank correlation coefficient (r = 0.418, p<0.01). All experiments were repeated at least three times. (TIF) [file pone.0070616.s005.tif]

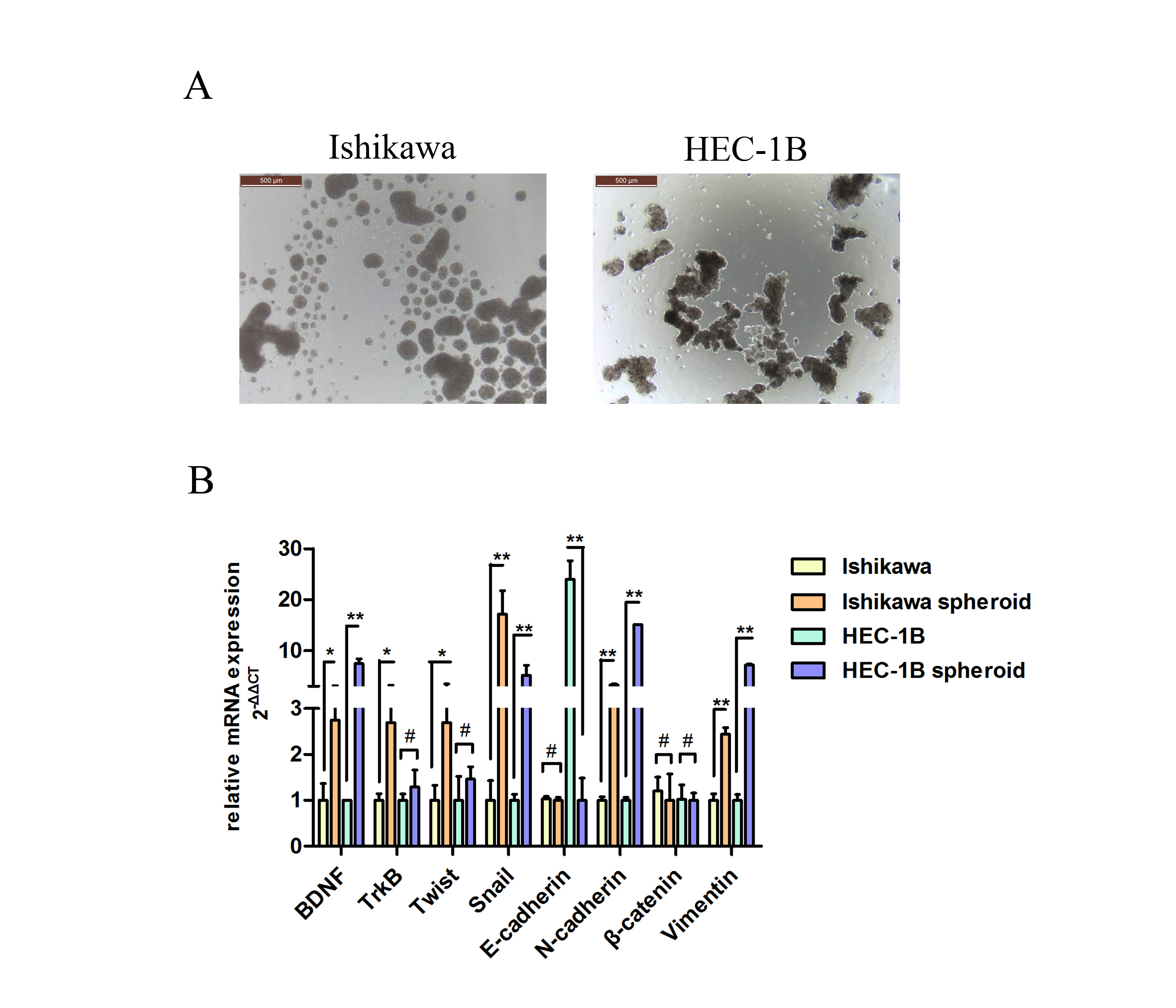

Supplement: Figure S6 — EMT markers are altered in anchorage-independent culture. A. Two kinds of EC cell aggregates in anchorage-independent culture (magnification, 40×). B. EMT markers in suspension and adherent cells were determined by qRT-PCR and assayed with the primers as mentioned. β-actin was included as a loading control. *p<0.05, **p<0.01; #, not significant. These experiments were repeated three times with similar results. (TIF) [file pone.0070616.s006.tif]
